# Formulation and Evaluation of the Antioxidant Activity of an Emulsion Containing a Commercial Green Tea Extract

**DOI:** 10.3390/molecules30010197

**Published:** 2025-01-06

**Authors:** Anna Sykuła, Izabela Janiak-Włodarczyk, Ireneusz Tomasz Kapusta

**Affiliations:** 1 Faculty of Biotechnology and Food Sciences, Institute of Natural Products and Cosmetics, Lodz University of Technology, Stefanowskiego 2/22, 90-537 Lodz, Poland; izabela.janiak@p.lodz.pl; 2 Department of Food Technology and Human Nutrition, College of Natural Sciences, University of Rzeszow, Zelwerowicza 4, 35-601 Rzeszow, Poland; ikapusta@ur.edu.pl

**Keywords:** green tea extract, antioxidant activity, emulsion

## Abstract

The addition of an extract to an emulsion is intended to improve its fragrance and care qualities. Green tea is a beverage known all over the world. It is tasty and has beneficial effects on human health due to its high polyphenol content. The compounds present in this variety of tea have also made it an interesting cosmetic ingredient. The polyphenols contained in green tea have antioxidant properties and can delay the ageing process in human skin. Various preparations with this ingredient can be found on the market—from creams to hair care products. Making one’s own cosmetics is also a trend. In the following study, three creams containing green tea extracts from three different manufacturers were prepared, and the total polyphenol (TP) contents, the phenolic profile of the extracts used and the antioxidant activity of these preparations were examined using two methods: DPPH^•^ and ABTS^•+^ cationic radicals. The study showed that the antioxidant activity of the glycerin–water extracts measured by the selected methods was higher than that of the oil extract. Among the creams, the product with green tea extract from Firm 2 (glycerin–water extract) showed the best antioxidant properties.

## 1. Introduction

Recipes for homemade natural cosmetics containing plant extracts can be found in many online sources today [1,2]. The users of these enriched emulsions are often of the opinion that they have a positive effect on the skin [3]. The fact that different extracts from the same plant can vary in their efficacy is not always obvious to consumers [4]. Not many people who use home cosmetics know in which solution green tea extract has the highest biological activity [5]. Prevention of skin aging is a very interesting topic that is generating intense discussions in scientific communities [6,7]. Antioxidants, which are becoming better known, are eagerly used in cosmetic preparations [7]. An example of a raw material showing antioxidant properties is green tea extract [5]. Its availability and price are causing growing interest in green tea as an ingredient in care products, not only for the skin, but also for hair [8].

Compounds with antioxidant properties found in green tea (*Camellia sinensis*) are primarily flavan-3-ols, such as (-)-epigallocatechin gallate (EGCG), (-)-epigallocatechin, (-)-epicatechin, or (-)-epicatechin gallate [9]. The content of these compounds in the dry weight of green tea can reach as much as 30% [5], with epigallocatechin gallate being the most abundant. An HPLC analysis of polyphenon 60, an extract from green tea that contains a mixture of polyphenolic compounds, in a study by Anna Masek and co-workers [10], indicated the content of individual phenolic compounds in the raw material.

Human skin is considered the largest organ of the human body, as it can account for up to 15% of the human mass [7]. Depending on the layer of the skin, the effect of the compounds contained in green tea extracts differs. In the stratum corneum, polyphenols have a predominantly antioxidant effect, while in deeper layers they show a protective effect against ultraviolet light, as well as having an effect on enzyme activity [5]. Polyphenols applied to the dermis can improve microcirculation and the condition of blood vessels, resulting in better oxygenation and nourishment of the skin, which is why they are often used in anti-cellulite products. Moreover, polyphenols have a protective effect against vitamin C, which is involved in collagen synthesis, and also have anti-inflammatory properties [11].

It is assumed that as little as 5% of green tea extract is an effective concentration. However, it is worth bearing in mind that catechins are light- and oxygen-sensitive compounds. In addition, they are hydrophilic compounds, therefore penetration of catechins through the skin may be limited [12].

The extraction efficiency and antioxidant activity depend on the extraction method and the solvent used for extraction [13]. The content of a variety of antioxidant compounds with differing chemical properties and their polarity either may or may not be soluble in a respective solvent [14]. Polar solvents are frequently applied for the extraction recovery of polyphenols from plant matrices. The most appropriate solvents are aqueous mixtures containing ethanol, methanol, acetone and ethyl acetate. Ethanol is known to be a good solvent for polyphenol extraction and is safe for human consumption. Methanol has generally been found to be more effective for the extraction of lower molecular weight polyphenols, whereas aqueous acetone is good for the extraction of higher molecular weight flavanols [15].

The aim of this study was to utilize the three most prevalent green tea extracts on the Polish market (two of which were glycerin–water extracts and one was an oil extract) in an emulsion, thereby demonstrating the effect of such extracts on the performance of cosmetics. The extracts were tested for total polyphenol content and polyphenol profile. After preparation, the creams were tested for antioxidant properties using two selected spectrophotometric methods—the DPPH^•^ radical method and the ABTS^•+^ cationic radical method.

## 2. Results

### 2.1. Form and INCI Composition of the Developed Emulsion

The selected form of emulsion was that of a cream. The selection of ingredients was guided by the parameters shown in Table 1.

The developed cream included the following ingredients: Aqua, Butyrospermum Parkii Shea Butter, Helianthus Annus Seed Oil, Cetearyl Olivate, Sorbitan Olivate, Hyaluronic Acid, Panthenol, Glycerin, Dehydroacetic Acid, Benzyl Alcohol, Camellia Sinensis Leaf Extract.

The prepared cream was an O/W emulsion. Preparation of the cream was begun by making a base, to which individual green tea extracts were added at a later stage. A base of 200 g was prepared. The contents of the ingredients of the cream base with a description of their function and name in the INCI system are presented in Table 2. The raw materials needed were purchased in 2023 on the Polish market via the internet. The selection of the extracts was guided by availability, price and product composition.

The prepared base was divided into four parts, to which three individual extracts were added in amounts such that the extract concentration by weight was about 1%. The names of the extracts (Firms 1, 2 and 3), their weights, quantities and pH values are shown in Table 3. The addition of green tea extract increased the pH value of the emulsion compared with the base emulsion.

### 2.2. Total Polyphenolic Content

**Figure 1:**
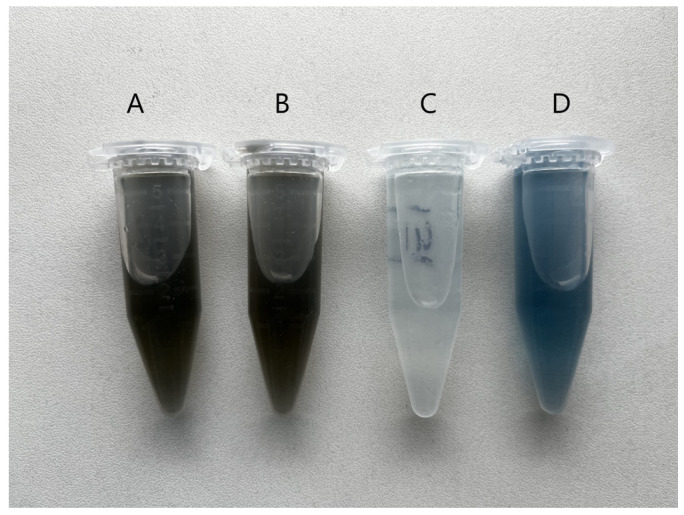
Visual appearance of samples during determination of total polyphenols content: (**A**) Extract from Firm 2 (glycerin–water); (**B**) extract from Firm 3 (glycerin–water); (**C**) extract from Firm 1 (oil sample); (**D**) gallic acid solution 1 mg/mL.

The Folin−Ciocalteu assay is a reference method for the quantification of total (poly)phenols in plant materials. The oxidizing F−C reagent reacts with reducing agents (antioxidants) to form a soluble, vividly blue complex [16]. This method was used to test all the extracts for their total polyphenol content. The characteristic color of the complex did not appear in the oil extract (Company 1, Figure 1C). Positive results (more brown, due to the original color of these extracts) in glycerol–water extracts with F–C reagent were observed directly in the vials (Figure 1A,B). The reference standard was a 1 mg/mL gallic acid solution with an F–C reagent (Figure 1D). The total polyphenol content of green tea extracts in the glycerin–water system was 73 ± 4 mg GAE/100 mL (Firm 2) and 57 ± 4 mg GAE/100 mL (Firm 3).

The oil extract did not give a positive result in this test. However, the glycerol–water extract did. Various combinations of solvents, hydrogen bond donors (ethylene glycol, glycerol, 1,2-butanediol, 1,4-butanediol, 2,3-butanediol, 1,3-butanediol, 1,6-hexanediol) with choline chloride (ChCl) have been optimized for the maximum recovery of polyphenols [17]. In order to determine the polyphenolic profile of the glycerol–water extracts (as they gave a positive result with the F–C reagent), their polyphenolic profile was analyzed. The oil extract was not used in this study.

### 2.3. HPLC Quantification of Polyphenol Content in Green Tea Extracts

Polyphenolic compounds in the green tea extracts were identified based on an analysis of characteristic spectral data—mass-to-charge ratio, *m*/*z*, and maximum absorption of radiation, which were compared with the available literature [18,19].

**Figure 2:**
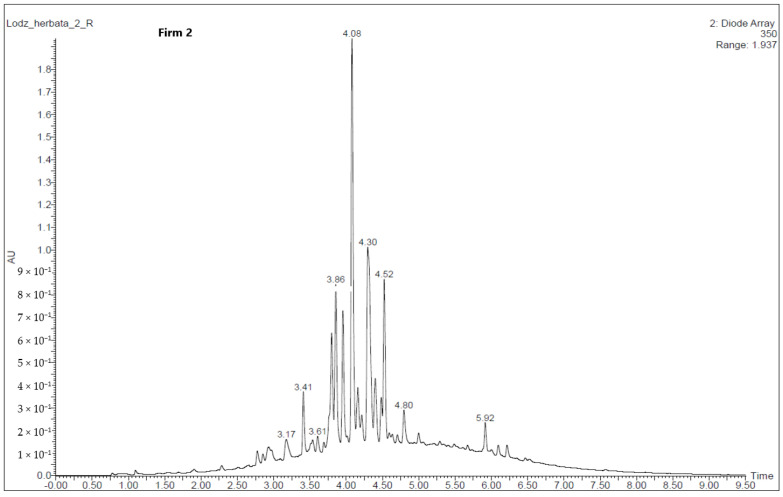
UPLC-MS chromatogram profile of the green tea glycerin–water extracts (Firm 2) at 350 nm. Peak number identification is displayed in Table 4.

**Figure 3:**
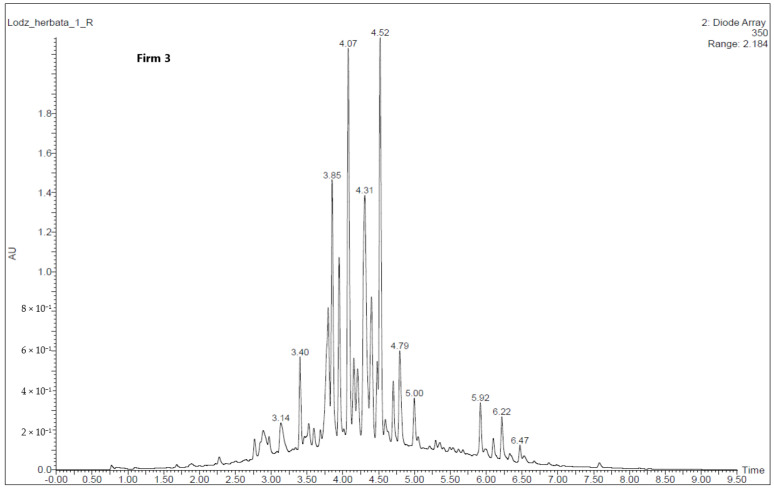
UPLC-MS chromatogram profile of the green tea glycerin–water extracts (Firm 3) at 350 nm. Peak number identification is displayed in Table 1.

Only two green tea glycerin–water extracts were analyzed by the UPLC-PDA-TQD-MS system. Results of qualitative analysis obtained by the UPLC with a PDA detector and FL are presented in Table 4 and Figure 2 and Figure 3. Based on the comparison of their retention times (Rt), MS and MS/MS data with those of standards, where available, and published data, 18 phenolic compounds belonging to the flavonols, flavan-3-ols and flavones were identified. The main classes of phenolic compounds in the analyzed green tea extract from Firm 2 were flavonols (59.6%) > flavones (32,8%) > flavan-3-ols (7.60%), from Firm 3—flavonols (66.2%) > flavones (24.4%) > flavan-3-ols (9.42%). The differences in individual polyphenol content between the two green tea extracts were highest between flavonols and flavones. According to Senanayake [9] green tea extract contains several polyphenolic components with antioxidant properties, but the predominant active components are the flavanol monomers known as catechins, where epigallocatechin-3-gallate and epicatechin-3-gallate are the most effective antioxidant compounds. In the case of glycerin–water extracts, this trend continues. The properties of the catechins in green tea depend on the manner in which the leaves are processed. Heating and fermentation can cause changes in the catechins, altering their properties [20]. The glycerol water extract from Firm 2 might be expected to have lower antioxidant activity based on the total polyphenol content.

### 2.4. Antioxidant Activity

#### 2.4.1. Extracts

In order to check the antioxidant activity of all of the extracts, two tests were carried out. One used the ABTS^•+^ radical and the other the DPPH^•^ radical.

Based on the antioxidant activity (AA%) results obtained using the cation radical ABTS^•+^, it was observed that the glycerol–water extracts of green tea showed a higher antioxidant capacity of 97.51–97.56% than the oil extract, with 63.78% (Table 5, *p* < 0.05).

Measuring antioxidant activity by the DPPH method, the AA% value for the Firm 1 extract was three times lower than for the other two extracts (Table 5, *p* < 0.05). This difference is greater than in the analysis involving ABTS.

Antioxidant activity was observed despite the negative TP for the oil extract from Firm 1. The DPPH radical showed the least activity. The activity value for the extract may be due to the nature of the oil itself. It is known that oils are capable of consuming free radicals. There are other antioxidants in vegetable oils that can scavenge ABTS and DPPH radicals in addition to polyphenols. These include vitamin E, carotenoids, phytosterols or synthetic phenolics [21].

#### 2.4.2. Emulsions

The cream samples after ethanol extraction showed a lower antioxidant activity using the cationic radical ABTS^•+^ compared with the three pure extracts. This can be clearly seen in Table 6. The addition of glycerol–water green tea extract from Firm 3 showed the greatest decrease in antioxidant activity (43.6%, the difference of the means is significant at the 0.05 level). Compared with the three pure extracts (Firm 1–3), the cream samples after acetone extraction showed a lower antioxidant activity using the cationic radical ABTS^•+^ (Table 6). Similar to the ethanol extraction of the emulsion, the greatest decrease in activity (68.44%, *p* < 0.05) was observed when the glycerol–water green tea extract from Firm 3 was used in the emulsion. A significant decrease in AA% (54.97%, *p* < 0.05) was also observed in the oil extract cream sample from Firm 1 (acetone). In conclusion, ABTS^•+^ testing of ethanolic emulsion samples enriched with green tea extract shows higher antioxidant activity than in acetone extracted emulsions. A significant decrease in the AA% was observed when the ethanol samples of the creams containing the three green tea extracts were analyzed by means of the DPPH radical—by 61.9% (*p* < 0.05) for Firm 3 and by 56.31% (*p* < 0.05) for Firm 2. Considering acetone samples of creams with three green tea extracts in the DPPH method, antioxidant activity values also decreased—62.82% (*p* < 0.05) for Firm 3 and 55.7% (*p* < 0.05) for Firm 2.

Concluding, in the measurement of antioxidant activity using the DPPH^•^ radical method, values similar for the two solvents used were obtained for the Firm 1 green tea extract cream. These values are quite low compared with the glycerin–water extracts tested, which may be due to the fact that the Firm 1 extract was an oil extract. The results for the method with ABTS^•+^ cationic radicals are very discrepant, which may indicate measurement errors (possibly due to the presence of a fat suspension in the test sample). A comparison of the antioxidant activity results for ethanol and acetone for the two methods used is shown in Table 5 and Table 6.

In the case of Firm 2’s green tea extract cream, a higher value for antioxidant activity was obtained using acetone as the solvent than ethanol, both when measured by the DPPH^•^ and ABTS^•+^ radical methods. This probably indicates a better separation of the components and antioxidant activity. The difference between solvents was insignificant. Table 6 shows a comparison of the antioxidant activity results for ethanol and acetone for the two methods used.

With regard to the cream with green tea extract of Firm 3, a higher value of antioxidant activity was obtained with ethanol as a solvent than with acetone, both when measured by the DPPH^•^ radical method and by the ABTS^•+^ radical method. The difference is not significant for the DPPH method. However, the large difference (as indicated by the large standard deviation for acetone) in the ABTS^•+^ method may be due to measurement error. Table 6 shows a comparison of the results of the antioxidant activity for ethanol and acetone for the two methods used.

As part of the testing of the emulsion, measurements were also taken on Trolox and a vitamin C cream from the chemist’s shop. The treatment of the cream was similar to the treatment of the emulsions in this work. Trolox showed a high level of activity in both methods (ABTS—96.80 ± 0.06% (ethanol); 96.85 ± 0.06% (acetone) and DPPH in ethanol 87.35 ± 0.42% and acetone 88.96 ± 0.1%). On the other hand, the vitamin C cream showed the best activity against the ABTS radical, both in ethanol (99.43 ± 0.73%) and in acetone (99.13 ± 0.44%).

## 3. Discussion

Polyphenols are powerful antioxidants that help to neutralize free radicals. Free radicals are unstable molecules that can damage skin cells. This can lead to premature ageing and other skin problems. Polyphenols help protect the skin from oxidative stress and damage by neutralizing free radicals [22] and can help reduce inflammation in the skin by acting as anti-inflammatories. This can be particularly beneficial for skin conditions such as acne, eczema and psoriasis. By reducing inflammation, polyphenols may soothe and relieve irritated skin [23]. Certain polyphenols can protect skin from UV damage. They can help prevent sunburn and reduce the risk of skin cancer by absorbing UV radiation and reducing the harmful effects of sun exposure [24]. Moreover, the skin’s hydration and elasticity can be improved by polyphenols. Polyphenols help maintain the skin’s moisture barrier by stimulating the production of collagen, a protein that keeps skin firm and elastic [7]. Some polyphenols can help with DNA repair. This is essential for maintaining healthy skin. They aid in the repair of damage caused by environmental factors such as UV radiation and pollution [22].

Green tea is a good source of polyphenols, including catechins, epicatechins, epigallocatechins, epicatechingallate, epigallocatechingallate, gallic acid, flavanoids, flavanols, and flavonols [17,25]. Therefore, green tea exhibits antioxidant activity and anti-carcinogenic, anti-obesity, anti-inflammatory, and antibacterial properties [26,27]. Tea has been confirmed to have a beneficial influence in the prevention of the risk of a number of medical conditions, such as cancer and cardiovascular disease [28,29,30].

On the cosmetics market in Poland and worldwide, there are various and widely available cosmetic products containing green tea extract additives. Consumers most often associate it as an ingredient in face creams, but it can also be found in shampoos and conditioners intended for use on hair. This reinforces the fact that it is an interesting and multifunctional ingredient, which is used in many cosmetic products.

The present study analyzed the antioxidant activity of cosmetic preparations containing extracts of green tea (*Camellia sinensis*). These extracts are a rich source of polyphenols—compounds of plant origin with high antioxidant capacity. A study by Samman et al. (2001) showed that the polyphenol content of green tea was 117.3 mg/g d.m., equivalent to 23.5 mg/100 mL for the conditions used [31]. A green tea infusion contains 111.6 mg GAE/100 mL of polyphenols, according to Kałwa and Wyrostek [32].

Due to its properties, green tea is gaining ever more interest among cosmetic manufacturers, resulting in a multitude of preparations containing its addition.

For research purposes, three cream samples containing green tea extracts from three different manufacturers were prepared. Two used extracts from Firms 2 and 3 were a glycerin–water mixture and one (Firm 1) was an oil extract. Their total polyphenol content using Folin−Ciocalteu reagent has been studied in order to determine whether the commercial extracts contain polyphenols in their composition. Only in the glycerol–water extracts were polyphenols confirmed. This effect was not seen in the oil extract. Only glycerol–water extracts were used to analyze polyphenolic compounds using UPLC-PDA-TQD-MS.

Two methods were used to test the antioxidant activity of extracts and prepared formulations—the ABTS^•+^ and the DPPH^•^. As part of the research for the paper, application tests were also carried out with four female probands to see how the prepared formulations affect the hydration and elasticity parameters of their skin.

Zych and Krzepiłko [33] compared the antioxidant capacity of the most popular antioxidants, including green tea, using the DPPH^•^ radicals. Among the compounds tested were gallic acid, caffeic acid, ascorbic acid, α-tocopherol, glutathione, and green tea infusion (Herbapol). The green tea infusion had the highest inhibitory capacity, 89.92 (±0.53), indicating that it had the greatest antioxidant properties of all the infusions and compounds tested. For the remaining samples (gallic acid, caffeic acid, α-tocopherol, ascorbic acid, glutathione), the inhibition values were as follows: 76.00 ± 0.72, 43.49 ± 3.77, 42.27 ± 3.09, 29.49 ± 0.88, 16.40 ± 0.38, respectively.

Zakaria and co-workers [34] investigated the antioxidant activity of green tea extracts using DPPH^•^ and ABTS^•+^ cation radicals. For this purpose, an aqueous extract of green tea was prepared. The study measured antioxidant activity for several other popular substances with antioxidant properties, such as resveratrol, moringa extract, amla extract, rhodiola extract, mango extract, pomegranate extract. The tea sample reduced ABTS^•+^ radicals at a level of 99.6 ± 0.03% and DPPH^•^ at 93.2 ± 0.4%. The high percentage of inhibition obtained for both methods allowed the researchers to classify the green tea extract as a substance with high antioxidant activity. In contrast, the cream with the extract showed lower antioxidant activity compared with the extract alone. The instability of the extract in the cosmetic formulation or the side reactions occurring between the cosmetic ingredients were considered to be the causes of this phenomenon. Furthermore, green tea extract was found to be pH-dependent and unstable in an alkaline environment [35].

A similar study was conducted by Gramza-Michalowska and co-workers [36]. Ethanolic green tea extracts were tested and antioxidant activity was measured using only the DPPH- radical method. In this study, a value of 95.4 ± 0.12% inhibition was obtained for the green tea extract, which is a high value, demonstrating the high antioxidant capacity of the test substance.

In the presented work all extracts showed reducing activity. The selection of solvents for the cream extracts was appropriate due to the fact that ethanol and acetone are highly efficient solvents in the extraction of phenolic compounds [37]. It was found that the acetone extract of the sample from Firm 2 had a higher hydrogen donation capacity in the DPPH (31.43%) and ABTS (80.56%) radical scavenging systems than the ethanol extracts. Acetone possesses the ability to dissolve hydrophilic and lipophilic compounds [38]. The solvent is very effective in the extraction of antioxidants such as phenols, being one of the main solvents used to extract this class of compounds from plants [39]. Certain properties of acetone, such as volatility, miscibility with polar and non-polar solvents, and moderate low toxicity to some microorganisms (e.g., *S. aureus* and *Pseudomonas aeruginosa*), render it a very suitable extraction solvent [40]. Regarding ethanol extraction, results are presented showing that ethanol extracts from Ivorian plants extract higher concentrations/quantities of phenols compared with acetone, water and methanol [41]. Another publication [42] has demonstrated that 80% ethanol shows the highest percentage of extraction yield, while 80% acetone extract shows the lowest extraction yield but the highest TPC, TFC and antioxidant activity of Opuntia stricta fruit. Saleem and co-workers [43] demonstrated that the maximum yield of catechins from green tea was obtained with a 50% ethanol–water system at 500 W power within 6 min of treatment.

Moreover, lower antioxidant activity was shown by cream extracts in the DPPH method. A comparative study by Martysiak-Zurowska et al. [44] on the assessment of the total antioxidant capacity of human milk using the ABTS and DPPH methods found that the latter showed lower sensitivity. The reason for this may probably be the limitations of the DPPH method, which include limited reactivity towards antioxidants and solubility in aqueous and organic solvents [45].

## 4. Conclusions

The main objective of the research presented in this work was to design and test the antioxidant activity of an emulsion (cream) containing green tea extract from different manufacturers. The main objective of the research presented in this thesis was to develop and test the antioxidant activity of an emulsion (cream) containing green tea extract from different manufacturers. Green tea extract is of great importance depending on the environment in which it is found. The oil extract does not show a total polyphenol content. However, the glycerol extracts do. Despite the absence of individual polyphenols, green tea oil extract was also observed in a study of antioxidant activity.

As part of the research for this study, creams containing green tea extracts from three different manufacturers (Firms 1–3) were formulated and prepared. Purchased extracts and prepared creams were subjected to antioxidant capacity analysis, using methods with ABTS cation radicals and DPPH radicals. The cream with the Firm 2 extract was found to have the highest antioxidant properties, which may indicate a higher content of polyphenols in the extract used to make the cream than in the other two extracts used.

## 5. Materials and Methods

### 5.1. Materials

Three Polish green tea extracts (two of them glycerin–water and one of them oil) were purchased via the internet. Glycerin, hyaluronic acid, D-panthenol, shea butter, emulsifier Olivem 1000, and preservative DHA + BA were purchased from ECOSPA s.c. (Warsaw, Poland). Sunflower oil was purchased from a supermarket. 2,2-Diphenyl-1-picrylhydrazyl (DPPH), 2,2′-Azino-bis(3-ethylbenzothiazoline-6-sulfonic acid) diammonium salt (ABTS), (±)-6-Hydroxy-2,5,7,8-tetramethylchromane-2-carboxylic acid (Trolox), potassium persulfate, and gallic acid were purchased from Sigma-Aldrich Co. (Poznań, Poland). Sodium carbonate, ethanol anhydrous 99.8% and acetone (both pure for basic analysis) were purchased from POCH (Gliwice, Poland). Folin–Ciocalteu reagent and sodium carbonate were purchased from CHEMPUR (Piekary Śląskie, Poland). All reagents were of analytical quality and were used without further purification.

### 5.2. Technology for Making Base Emulsion

Water, glycerin, hyaluronic acid and D-panthenol were weighed out in a 300 mL beaker and placed on a magnetic stirrer. The stirring mixture was heated to 75 °C. In a second 300 mL beaker, shea butter, sunflower oil and the emulsifier Olivem 1000 were weighed out. The beaker was placed on a magnetic stirrer. The stirring mixture was heated to 75 °C. The contents of the two phases were combined when identical temperatures were reached. The oil phase was carefully transferred to the aqueous phase. Their homogenization was carried out using an IKA T50 Digital ULTRA-TURRAX^®^ homogenizer at 1000 rpm for approximately 10 min. The emulsion beaker was cooled to 30 °C. A preservative was added to the emulsion and re-homogenization was carried out (5 min, 1000 rpm). The resulting emulsion was divided into 4 parts with known masses (Table 3). One green tea extract from three selected manufacturers was introduced into each part. After the addition of the extract, a short homogenization was carried out (5 min, 1000 rpm). The pH and electrical conductivity [µS/cm] using laboratory pH/conductivity/salinity meter CPC-505 (Elmetron, Zabrze, Poland) of the prepared emulsions were measured. Their values were as follows: Emulsion 1: 5.96 (115.10 µS/cm), Emulsion 2: 5.74 (111.78 µS/cm), Emulsion 3: 5.96 (114.94 µS/cm), Emulsion 4: 5.23 (110.06 µS/cm).

The prepared creams were transferred to sterile containers.

### 5.3. Total Polyphenol (TP) Contents

The analysis of TP content in green tea extracts was based on the method published by Singleton and Rossi [46]. The analysis procedure was as follows: Briefly, in a 4 mL solution of 50 µL glycerin–water green tea extract was diluted and added 100 µL Folin–Ciocalteu reagent. The solution was incubated for 3 min in the dark. After this time, 1 mL of a 10% sodium carbonate solution was added. The whole was mixed and incubated for 60 min in a dark place. Then the specific absorbance at 760 nm was measured on a Hewlett-Packard 8453 spectrophotometer running 845× UV–Visible ChemStation v. B.05.04 Software (Agilent, Mulgrave, VIC, Australia). Total polyphenol content was determined against a standard curve of appropriately prepared solutions with 1 mg/mL gallic acid stock.

### 5.4. Phenolic Profile of Extracts Used

Determination of polyphenolic compounds was carried out using the ultra-performance liquid chromatography (UPLC) Waters ACQUITY system (Waters, Milford, MA, USA). The UPLC system was equipped with a binary pump manager, column manager, sample manager, photodiode array (PDA) detector and tandem quadrupole mass spectrometer (TQD) with an electrospray ionization (ESI) source. Separation of polyphenols was performed using a 1.7 µm, 100 mm × 2.1 mm UPLC BEH RP C18 column (Waters, USA). For the investigation, the mobile phase consisted of 0.1% formic acid in water, *v*/*v* (solvent A) and 0.1% formic acid in 40% acetonitrile, *v*/*v* (solvent were used). The flow rate was kept constant at 0.35 mL/min for a total run time of 8 min. The system was run with the following gradient program: from 0 min 5% B, from 0 to 8 min linear to 100% B and from 8 to 9.5 min for washing and back to initial conditions. The injection volume of the samples was 5 µL, and the column was supported at 50 °C. The following TQD parameters were used: cone voltage of 30 V, capillary voltage of 3500 V, source and desolvation temperature 120 °C and 350 °C, respectively, and desolvation gas flow rate of 800 L/h. Characterization of the individual polyphenolic compounds was performed on the basis of the retention time, mass-to-charge ratio, fragment ions and comparison of data obtained with commercial standards and literature findings. Obtained data were processed in Waters MassLynx v.4.1 software (Waters, Milford, MA, USA).

### 5.5. Determination of Antioxidant Activity

Analysis of the antioxidant properties using the cation radical ABTS^•+^ and DPPH^•^ in creams with green tea extracts was performed using two solvents, acetone and ethanol. Approximately 1 g of each cream was weighed and 2 mL of solvent was added. The samples were sonified twice for 15 min. They were then centrifuged (15 min, 10,000 rpm). Each sample was filtered using a cellulose acetate filter (0.45 μm). The filtrate was transferred into clean Eppendorf tubes in triplicate.

All analyses were undertaken at least in triplicate, and these values were then presented as average values along with their standard derivations.

#### 5.5.1. Using the Cation Radical ABTS^•+^

The antioxidant activity was determined according to the modified method of Re and coworkers [47]. A stock solution was prepared to analyze the antioxidant capacity of the samples tested using the cation radical ABTS^•+^. An amount of 20 mL of a 2.5 mM ABTS solution and a 1 mM potassium persulphate solution were mixed. The prepared solution, whose role will be to act as a radical, was incubated at room temperature and in a dark place for 18 h. After the indicated time, the ABTS^•+^ cation radical solution was diluted with ethanol to give an absorbance of 0.7 ± 0.02 at 734 nm. To measure antioxidant properties in selected green tea extracts using the cation radical ABTS^•+^, 21 μL of each extract was taken in three series into clean Eppendorf tubes and supplemented with 79 μL of distilled water. The samples were sonicated twice (15 min). Prior to the measurements, 2 mL of ABTS^•+^ solution was added to each of the prepared samples containing 100 μL of each extract. Absorbance measurements were taken after 5 min of incubation. Absorbance measurements were carried out on a Hewlett-Packard 8453 spectrophotometer running an 845_ UV–Visible ChemStation v. B.05.04 Software (Agilent, Mulgrave, VIC, Australia). The wavelength for the measurements was 745 nm. Ethanol was chosen as the background.

Analysis of the antioxidant properties using the cation radical ABTS^•+^ in creams with green tea extracts was performed using two solvents, acetone and ethanol. Approximately 1 g of each cream was weighed and 2 mL of solvent was added. The samples were sonified twice for 15 min. They were then centrifuged (15 min, 10 000 rpm). Each sample was filtered using a cellulose acetate filter (0.45 μm). The filtrate was transferred in a volume of 100 μL to clean Eppendorf tubes in triplicate. Prior to the measurements, 2 mL of ABTS^•+^ solution was added to each of the prepared samples containing 100 μL of each extract/sample cream. Absorbance measurements were taken after 5 min of incubation.

As part of the testing of the emulsion, measurements were also taken on Trolox at a concentration 0.1 mg/mL and on a chemist’s vitamin C cream. The treatment of the cream was similar to that of the emulsions.

The radical scavenging activity, antioxidant activity (AA%) in the ABTS^•+^ method was calculated using the following formula [34]:

(1) $$AA\left[\%\right]=\left(1-\frac{A_{sample}}{A_{control}}\right)\cdot 100\%$$

where *A_sample_* is the absorbance of the sample and *A_control_* is the absorbance of ABTS^•+^ solution.

#### 5.5.2. Using the Cation Radical DPPH

The antioxidant activity was determined according to the modified method of Brand-Wiliams et al. using the synthetic radical DPPH^•^ [48].

To measure antioxidant properties in selected green tea extracts using the cation radical DPPH, 21 μL of each extract was taken in three series into clean Eppendorf tubes and supplemented with 79 μL of distilled water. Absorbance measurements were carried out on a Hewlett-Packard 8453 spectrophotometer running 845_ UV–Visible ChemStation v. B.05.04 Software (Agilent, Mulgrave, VIC, Australia). The samples were sonicated twice (15 min). The wavelength for the measurements was 517 nm. Ethanol was chosen as the background. Prior to the measurements, 3.9 mL of DPPH- solution was added to each of the prepared samples containing 100 μL of the individual extract. Absorbance measurements were taken after 10 min of incubation.

As part of the testing of the emulsion, measurements were also taken on Trolox at a concentration 0.1 mg/mL and on a chemist’s vitamin C cream. The treatment of the cream was similar to that of the emulsions.

The radical scavenging activity, antioxidant activity (AA%) in the DPPH- method was calculated using the following formula [34]:

(2) $$AA\left[\%\right]=\left(1-\frac{A_{sample}}{A_{control}}\right)\cdot 100\%$$

where *A_sample_* is the absorbance of sample and *A_control_* is the absorbance of DPPH^•^ solution.

## 6. Statistical Analysis

Data statistics were calculated using Excel 365 (Microsoft Corp.; Redmond, WA, USA) and OriginPro 2018 64-bit (OriginLab Corp.; Northampton, MA, USA) programs. The results are presented as arithmetic means with standard deviations. Comparisons of the results found via analysis of the sample were made using one sample *t*-Test comparisons between means and were made using Tukey’s test for probability values *p* < 0.05. A one-way analysis of variance (ANOVA) with Tukey’s post hoc tests was performed to determine the significance of differences at *p* < 0.05.

## Figures and Tables

**Table 1:** Percentage of individual phases in the prepared cream, % by weight.

Emulsifier Content% by Weight	Oil Phase			Aqueous Phase	Other Resources	
	20–30				Active	Preservative
8	**Liquid Ingredients**		**Constant Ingredients**	Up to 100	q.s.	q.s.
	**Oils**	**Emollients**				
	0–10	20–30	3–5			

**Table 2:** Raw materials used to make the cream base, their quantity, function and name in the INCI system.

No.	Raw Materials	Content		Function	INCI
		I [g]	II [%]		
1.	Water	120.72	60.25	Solvent	Aqua
2.	Shea butter	20.57	10.27	Emollient	Butyrospermum Parkii Shea Butter
3.	Sunflower oil	20.40	10.18	Emollient	Helianthus Annus Seed Oil
4.	Olivem 1000	16.19	8.08	Emulsifier	Cetearyl Olivate, Sorbitan Olivate
5.	Triple hyaluronic acid 3% solution	10.95	5.46	Active ingredient, moisturizes skin	Hyaluronic Acid
6.	D-Panthenol	5.18	2.59	Active ingredient, relieves irritation	Panthenol
7.	Glycerin	5.16	2.58	Active ingredient, humectant	Glycerin
8.	Preservative DHA + BA (dehydroacetic acid, benzyl alcohol)	1.20	0.60	Preservative	Dehydroacetic Acid, Benzyl Alcohol

**Table 3:** Mass and percentage of green tea extracts in the different cream versions and their pH values.

Name of Sample	Mass of Sample [g]	Addition of Extract	Mass of Extract [g]	Percentage of Extract in Emulsion [%]	pH
Emulsion 1	46.901	Firm 1 (oil)	0.446	0.951	5.96
Emulsion 2	55.071	Firm 2 (glycerin–water)	0.607	1.103	5.74
Emulsion 3	52.703	Firm 3 (glycerin–water)	0.609	1.155	5.88
Emulsion 4	45.325	Without extract	-	-	5.23

**Table 4:** Polyphenolic profile in the tested green tea extracts [mg/100 mL] identified by UPLC-PDA-TQD-MS.

Compound	R_t_ [min]	MS [*m*/*z*]^-^	MS/MS [*m*/*z*]	λ_max_ [nm]	Extract Firm 2	Extract Firm 3
(-)Epigalocatechin 3-*O*-gallate	3.41	457	169	272	97.83 ± 1.52	153.24 ± 2.86
Apigenin 6-*C*-xyloside-8-*C*-glucoside	3.79	563	431, 269	269, 342	266.65 ± 4.69	480.18 ± 24.83
Myricetin-3-*O*-glucoside	3.86	479	317	255, 352	317.56 ± 11.99	542.94 ± 21.55
Quercetin 3-*O*-rutinoside-7-*O*-glucoside	3.95	771	609, 301	256, 352	233.11 ± 7.05	328.08 ± 5.11
Apigenin 6-*C*-glucoside-8-*C*-glucoside	4.07	593	431, 269	267, 347	753.64 ± 9.65	826.45 ± 14.53
Quercetin 3-*O*-rutinoside-7-*O*-glucoside	4.15	771	609, 301	255, 352	114.41 ± 5.45	166.47 ± 6.29
Quercetin 3-*O*-rutinoside-rhamnoside	4.21	755	301	255, 355	52.25 ± 0.11	152.76 ± 4.62
Quercetin 3-*O*-rutinoside-7-*O*-rhamnoside	4.31	755	609, 301	255, 352	616.18 ± 12.31	806.89 ± 10.34
(-)Epicatechin-3-*O*-gallate	4.43	441	169	272	140.12 ± 7.96	351.06 ± 16.72
Kaempferol 3-*O*-rutinoside-glucoside	4.48	755	285	267, 350	62.97 ± 0.19	117.13 ± 0.26
Kaempferol 3-O-rutinoside-7-*O*-glucoside	4.52	755	593, 285	264, 346	275.21 ± 1.93	776.95 ± 15.52
Kaempferol 3-*O*-rutinoside-7-*O*-rhamnoside	4.70	739	593, 285	267, 350	11.91 ± 0.14	109.81 ± 6.24
Kaempferol 3-*O*-rutinoside	4.80	593	285	267, 345	65.25 ± 1.41	203.90 ± 0.60
Kaempferol 3-*O*-glucoside	4.99	447	285	267, 350	15.17 ± 0.16	95.72 ± 0.67
Kaempferol 3-*O*-p-coumaroyl-rutinoside-7-*O*-rhamnoside-glucoside	5.92	1049	593, 285	269, 320	47.29 ± 0.49	91.96 ± 1.12
Kaempferol 3-*O*-p-coumarylo-rutinoside-7-*O*-rhamnoside	6.10	887	593, 285	269, 322	15.61 ± 0.40	35.57 ± 0.77
Kaempferol 3-*O*-p-coumarylo-rutinoside-7-*O*-dirhamnoside (I)	6.22	1033	593, 285	267, 320	22.48 ± 0.91	91.05 ± 0.96
Kaempferol 3-*O*-p-coumarylo-rutinoside-7-*O*-dirhamnoside (II)	6.47	1033	593, 285	267, 320	4.35 ± 0.19	25.23 ± 0.26
Σ Total PC					3111.99 ± 64.98	5355.39 ± 128.58

**Table 5:** Antioxidant activity of green tea extracts made by the ABTS and DPPH methods.

Manufacture	ABTS AA [% ± SD]	DPPH AA [% ± SD]	Solution Appearance
Firm 1	63.78 ± 7.13 *	30.18 ± 12.84 *	transparent
Firm 2	97.56 ± 0.78	87.13 ± 1.36	
Firm 3	97.51 ± 2.33	86.59 ± 1.29	

Results presented as mean values of three replicates with standard deviations. * Significantly different from the other values (*p* < 0.05).

**Table 6:** Antioxidant activity made by the ABTS and DPPH methods in emulsion with and without green tea extracts by extraction in ethanol and acetone.

Solvent	Manufacture	ABTS AA [% ± SD]	Solution Appearance	DPPH AA [% ± SD]	Solution Appearance
Ethanol	Firm 1	59.65 ± 18.67	Transparent	19.53 ± 3.39	Transparent
	Firm 2	77.43 ± 5.64		30.82 ± 1.36	
	Firm 3	53.91 ± 3.96		24.70 ± 0.36	
	Emulsion without green tea extract	12.69 ± 1.45		13.72 ± 1.45	
	Vitamin C cream	99.13 ± 0.44		30.38 ± 1.68	
	Trolox	95.93 ± 0.24		87.35 ± 0.42	
Acetone	Firm 1	8.81 ± 4.09	Obscure	18.90 ± 3.71	Transparent
	Firm 2	80.56 ± 13.47		31.43 ± 2.59	
	Firm 3	29.07 ± 7.20		23.77 ± 2.61	
	Emulsion without green tea extract	−73.62 ± 52.02		14.33 ± 1.55	
	Vitamin C cream	99.43 ± 0.73		30.06 ± 2.18	
	Trolox	95.95 ± 0.04		88.96 ± 0.1	

Results presented as mean values of three replicates. All values are significantly different at *p* < 0.05.

## Data Availability

Data are contained within the article.
